# Determining oncogenic patterns and cancer predisposition through the transcriptomic profile in Mitchell–Riley syndrome with heterotopic gastric mucosa and duodenal atresia: a case report

**DOI:** 10.1186/s13023-021-02093-9

**Published:** 2021-10-29

**Authors:** Valeria Calcaterra, Luigi Chiricosta, Emanuela Mazzon, Agnese Gugnandolo, Daniele Alberti, Luciano Maestri, Milena Meroni, Elettra Vestri, Elvira Verduci, Dario Dilillo, Gianvincenzo Zuccotti, Gloria Pelizzo

**Affiliations:** 1 Pediatrics and Adolescentology Unit, Department of Internal Medicine, University of Pavia, Pavia, Italy; 2 Pediatric Department, Children’s Hospital “Vittore Buzzi”, Milan, Italy; 3 IRCCS Centro Neurolesi “Bonino-Pulejo”, Messina, Italy; 4 Pediatric Surgery Department, “Spedali Civili” Children’s Hospital, Brescia, Italy; 5 Pediatric Surgery Department, Children’s Hospital “Vittore Buzzi”, Via Lodovico Castelvetro n.32, 20154 Milan, Italy; 6 Department of Health Sciences, University of Milano, Milan, Italy; 7 Department of Biomedical and Clinical Science “L. Sacco”, University of Milano, Milan, Italy

**Keywords:** Heterotopic gastric mucosa, Duodenal atresia, Mitchell–Riley syndrome, RFX6, Transcriptomic profile, Case report

## Abstract

**Background:**

Homozygous mutations in the transcription factor *RFX6* are the cause of the Mitchell–Riley syndrome (MRS) associating neonatal diabetes, congenital digestive system, such as biliary atresia, pancreatic hypoplasia, duodenal and/or jejunal atresia, intestinal malrotation, gallbladder aplasia, cholestasis. A constitutive inactivation of RFX6 leads also to gastric heterotopia. Application of RNA-seq in human diseases may help to better understand pathogenic mechanism of diseases and to predict the risk of developing chronic disorders and personalizing their prevention and treatment. We evaluated oncogenic patterns and cancer predisposition using the transcriptomic profile in a case of MRS with neonatal diabetes, duodenal atresia, and extensive intestinal tract gastric heterotopia.

**Results:**

We signalled the interactors of *RFX6* with other up and downregulated genes, that may be interested in severity of diabetic condition, in multi-organs impairment and cancer predisposition. Furthermore, several dysregulated genes are involved in biological processes that can lead to promote cancer including “Evading apoptosis” (*BAD, BBC3, EGF, FGFR2, FLT3LG, HMOX1, HRAS, IFNAR2, IGF1R, IL12RB1, IL13RA1, IL15, IL2RB, IL2RG, IL6R, KEAP1, MGST1, PDGFA, PDGFRB, PIK3R3, RALB, RALGDS, RASSF1, SOS1, TGFA, TXNRD3)*, “Proliferation” (*APC, BRAF, CCND2, CCND3, CCNE2, FGFR2, FLT3LG, FZD1, FZD6, HMOX1, HRAS, IGF1R, KEAP1, LRP6, MAPK3, MGST1, PDGFA, PDGFB, PDGFRB, RB1, SOS1, TGFA, TXNRD3, WNT10B*), “Sustained angiogenesis” (*BRAF, FGFR2, FLT3LG, HRAS, IGF1R, JAG1, MAPK3, NOTCH2, PDGFA, PDGFB, PDGFRB, SOS1, TGFA, TGFB1),* “Genomic instability” (*BAD, BBC3*) and “Insensitivity to anti-growth signals” (*SMAD2, TGFB1*). We also inspected the signalings and their related genes in cancer, such as “PI3K signaling”, “ERK signaling”, “JAK-STAT signaling”, “Calcium signaling”, “Other RAS signaling”, “WNT signaling”.

**Conclusions:**

In our MRS patient, we signaled the interactors of *RFX6* with other up- and downregulated genes that may be related to severe diabetic condition, multi-organ impairment, and cancer predisposition. Notably, many dysregulated genes may lead to triggering carcinogenesis. The possibility of the patient developing cancer degeneration in heterotopic gastric mucosa and/or additional long-term tumoral sequelae is not excluded. Personalized prevention and treatment strategies should be proposed.

## Background

Mitchell–Riley syndrome (MRS) is an autosomal recessive disorder caused by mutations in the *RFX6* gene in which a combination of neonatal diabetes mellitus and congenital gastrointestinal defects—such as atresia, stenosis or malrotation of the small intestine, gallbladder hypoplasia or agenesis, intrahepatic or extrahepatic ductal atresia, or hypoplastic or annular pancreas—occur [1–6]. In a limited number of patients with MRS, heterotopic jejunal gastric mucosa, including in the duodenal and jejunal tract, has been described [2, 7, 8]. Even though the link between RFX6 and heterotopic gastric mucosa has not been extensively studied, as reported by Piccand et al. [9], RFX6 is required for the maintenance of intestinal cell identity and the constitutive inactivation of RFX6 leads to gastric heterotopia.

Heterotopic gastric mucosa is a pathological condition that has rarely been reported on and consists of ectopic gastric mucosa. It can be discovered anywhere throughout the gastrointestinal tract and may be asymptomatic or present with intussusception, obstruction, pain, bleeding, ulceration, or perforation. The association between intestinal tract gastric heterotopia and carcinogenesis is controversial [10–15]. Heterotopic gastric mucosa grow in the submucosa. Surface mucosa are vulnerable to repetitive erosion and regeneration, and these could be the cause of aberrations in the gastric mucosa and the development of cancer degeneration at the surface mucosa [16].

The pathogenesis of congenital anomalies (CAs), such as duodenal atresia associated with intestinal gastric heterotopia, has not been fully elucidated. Due to interactions between genes and the environment, during the organogenesis phase a modification of normal embryo-fetal development may occur [17]. The memory of the insult will be retained by the organism and may result in pathology later on, such as an increased risk of noncommunicable diseases (NCDs) and cancer predisposition [18, 19].

The recent application of scRNA-seq in human diseases may help us to better understand the pathogenic mechanisms of diseases [18]. The identification of gene expression patterns may be useful for predicting the risk of developing chronic diseases and help to personalize prevention and treatment methods [18–22].

We evaluated oncogenic patterns and cancer predisposition using the transcriptomic profile in a case of Mitchell–Riley syndrome with neonatal diabetes, duodenal atresia, and extensive intestinal tract gastric heterotopia, including the duodenum and the jejunum. Sequencing could aid in the implementation of personalized prevention and treatment strategies.

## Results

The analysis of DEGs revealed that 4834 transcripts passed all the filters in MRS. Among them, 2202 transcripts had a fold change higher than 2, while 2632 had one lower than − 2. The mean of the distribution of the fold change in the upregulated genes is 4.35+/− 2.18, while that in the downregulated genes is − 4.33+/− 1.92.

**Figure 1.**
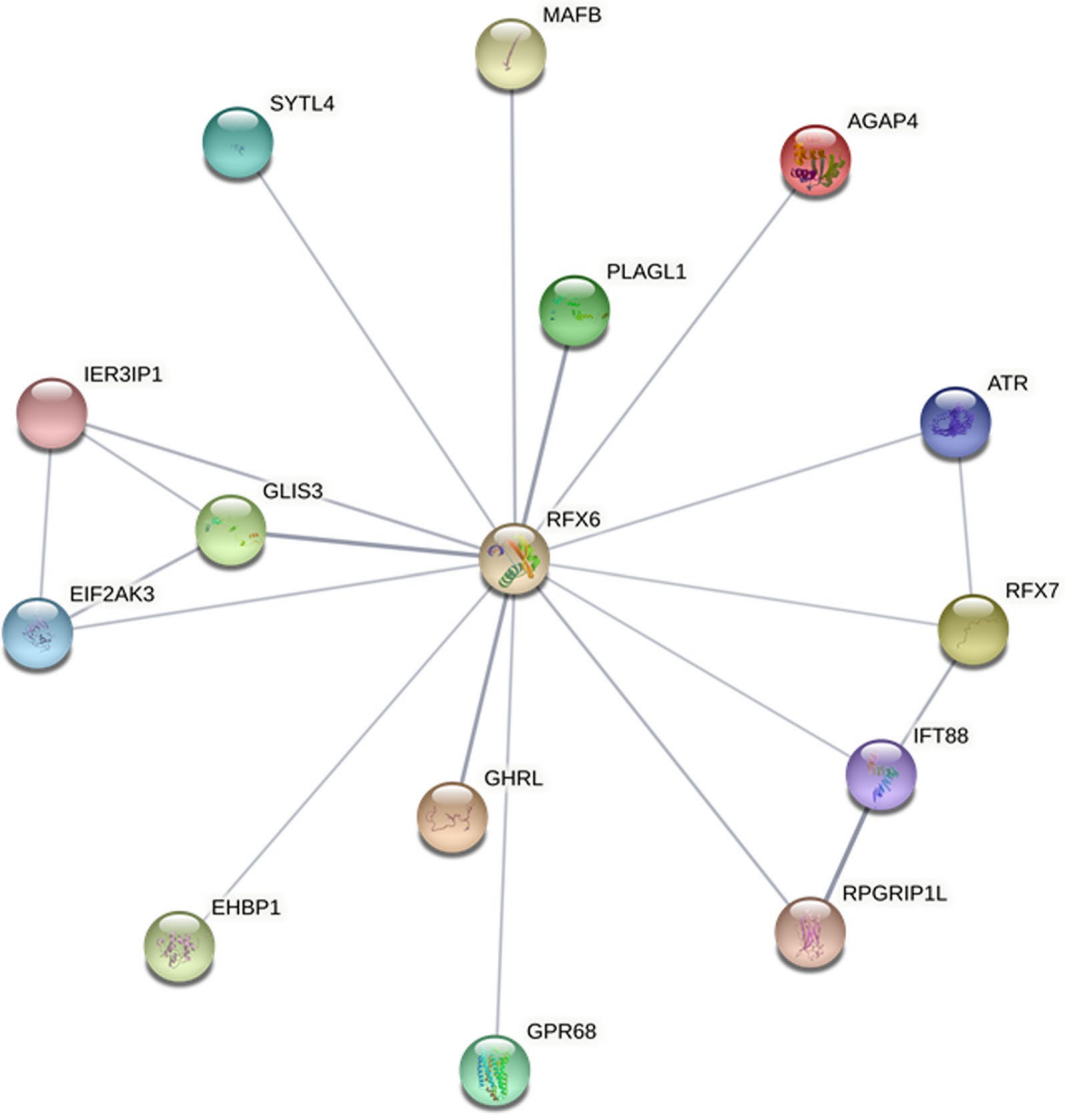
Interactors of *RFX6* that are deregulated in our analysis obtained with STRING. Ignoring the construction *RFX6*, the genes *EIF2AK3, IER3IP1, GLIS3, RFX7* and *IFT88* have 3 as the degree that is the highest value in the network

MRS is known to be associated with mutations in the *RFX6* gene. For this reason, we inspected the closest interactors of RFX6 deregulated in our analysis. We observed nine genes with upregulated fold changes (*AGAP4, ATR, EHBP1, EIF2AK3, GLIS3, IFT88, RFX7, RPGRIP1L, SYTL4*) and five genes with downregulated fold changes (*GHRL, GPR68, IER3IP1, MAFB, PLAGL1*), as reported in the fold change column of Table 1. Additionally, the table shows for each gene the transcripts’ mean counts for the control or MRS group obtained after DESeq2 normalization. Among these interactors, *EIF2AK3, GLIS3, IER3IP1,* and *PLAGL1* are linked to Diabetes mellitus in Swiss-Prot. In addition, *GPR68* is associated with tumor suppressor and *MAFB* is associated with proto-oncogene and tumor suppressor activities. In Figure 1 the 14 DEG interactors of *RFX6* are plotted. Notably, *EIF2AK3*, *IER3IP1*, *GLIS3*, *RFX7*, and *IFT88* were present at the highest degree (3).

**Table 1.** DEGs interactors of *RFX6* in our analysis

Gene symbol	Gene name	Control mean counts	MRS mean counts	Fold change	q-value
*AGAP4*	ArfGAP with GTPase domain, ankyrin repeat and PH domain 4	97.37	4969.96	5.67	6.96e−24
*ATR*	ATR serine/threonine kinase	1005.56	5420.26	2.43	4.94e−12
*EHBP1*	EH domain binding protein 1	316.07	2251.49	2.84	1.93e−20
*EIF2AK3*	Eukaryotic translation initiation factor 2 alpha kinase 3	761.30	4152.75	2.45	6.13e−14
*GHRL*	Ghrelin and obestatin prepropeptide	308.44	16.68	− 4.21	1.58e−02
*GLIS3*	GLIS family zinc finger 3	13.28	200.13	3.88	2.23e−04
*GPR68*	G protein-coupled receptor 68	806.28	0	− 8.04	6.51e−04
*IER3IP1*	Immediate early response 3 interacting protein 1	303.11	0	− 6.63	5.38e−03
*IFT88*	Intraflagellar transport 88	192.00	1250.83	2.70	4.18e−11
*MAFB*	MAF bZIP transcription factor B	1639.27	216.81	− 2.92	3.19e−03
*PLAGL1*	PLAG1 like zinc finger 1	963.13	166.78	− 2.53	1.11e−04
*RFX7*	Regulatory factor X7	794.65	3635.74	2.19	2.45e−07
*RPGRIP1L*	RPGRIP1 like	55.03	300.20	2.42	2.47e−04
*SYTL4*	Synaptotagmin like 4	24.76	200.13	3.01	6.69e−03

We highlighted the transcripts mean counts obtained after DESeq2 normalization, the fold changes computed as log_2_ (MRS mean counts/control mean counts) and the q-value for each of the DEGs in our analysis that interacts with RFX6 in STRING. All values are rounded to the second decimal digit

**Figure 2.**
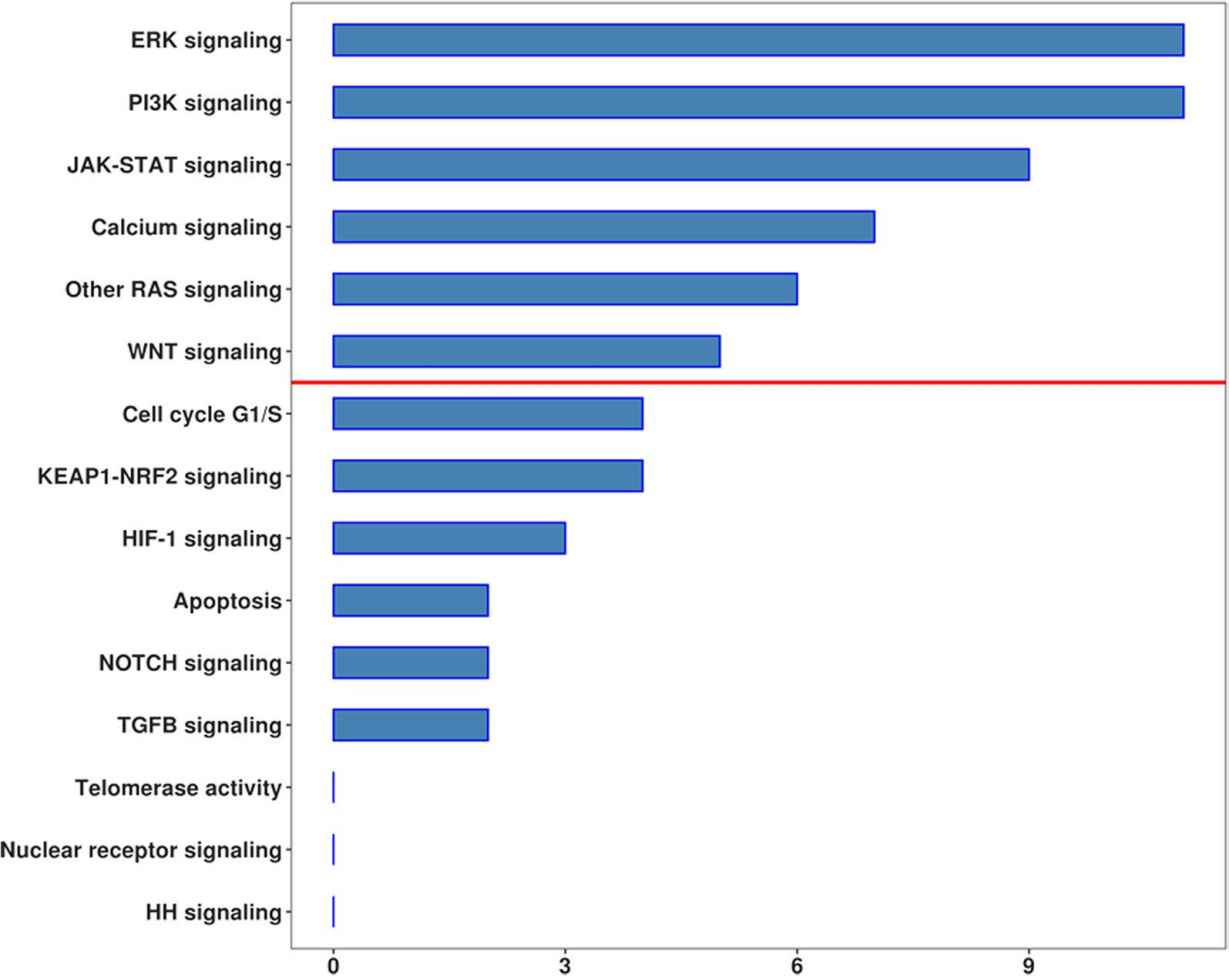
Number of DEGs in the processes identified inside the KEGG map “Pathways in cancer”. The red line represent the median value so that “Evading apoptosis”, “Proliferation”, “Sustained angiogenesis”, “Genomic instability” and “Insensitivity to anti-growth signals” have a major implication in our study

Among the DEGs highlighted in our analysis, 100 were characterized in several processes in the KEGG map “pathways in cancer” (Figure 2). We observed (Table 2) that the major implications occurred for “evading apoptosis” (*BAD, BBC3, EGF, FGFR2, FLT3LG, HMOX1, HRAS, IFNAR2, IGF1R, IL12RB1, IL13RA1, IL15, IL2RB, IL2RG, IL6R, KEAP1, MGST1, PDGFA, PDGFRB, PIK3R3, RALB, RALGDS, RASSF1, SOS1, TGFA, TXNRD3)*, “proliferation” (*APC, BRAF, CCND2, CCND3, CCNE2, FGFR2, FLT3LG, FZD1, FZD6, HMOX1, HRAS, IGF1R, KEAP1, LRP6, MAPK3, MGST1, PDGFA, PDGFB, PDGFRB, RB1, SOS1, TGFA, TXNRD3, WNT10B*), “sustained angiogenesis” (*BRAF, FGFR2, FLT3LG, HRAS, IGF1R, JAG1, MAPK3, NOTCH2, PDGFA, PDGFB, PDGFRB, SOS1, TGFA, TGFB1),* “genomic instability” (*BAD, BBC3*) and “insensitivity to anti-growth signals” (*SMAD2, TGFB1*).

**Table 2.** DEGs inside the KEGG map “Pathways in cancer” identified in the most implicated processes

Biological implication	Gene symbol	Gene name	Control mean counts	MRS mean counts	Fold change	q-value
Evading apoptosis	*BAD*	BCL2 associated agonist of cell death	826.17	116.74	− 2.82	5.74e−05
	*BBC3*	BCL2 binding component 3	1275.40	66.71	− 4.26	5.98e−06
	*EGF*	Epidermal growth factor	62.50	700.46	3.49	2.01e−04
	*FGFR2*	Fibroblast growth factor receptor 2	85.98	950.63	3.47	4.66e−03
	*FLT3LG*	fms related tyrosine kinase 3 ligand	92.37	0	− 4.90	4.48e−02
	*HMOX1*	heme oxygenase 1	3398.05	733.82	− 2.21	1.22e−03
	*HRAS*	HRas proto− oncogene, GTPase	460.16	83.39	− 2.46	2.96e−03
	*IFNAR2*	Interferon alpha and beta receptor subunit 2	2464.19	83.39	− 4.89	6.69e−11
	*IGF1R*	Insulin like growth factor 1 receptor	2771.65	11591.01	2.06	1.07e−03
	*IL12RB1*	Interleukin 12 receptor subunit beta 1	2509.33	617.08	− 2.02	1.76e−05
	*IL13RA1*	Interleukin 13 receptor subunit alpha 1	5850.09	1184.12	− 2.30	2.81e−05
	*IL15*	Interleukin 15	240.69	50.03	− 2.26	4.34e−02
	*IL2RB*	Interleukin 2 receptor subunit beta	8781.96	733.82	− 3.58	8.80e−08
	*IL2RG*	Interleukin 2 receptor subunit gamma	6894.04	33.36	− 7.69	1.12e−11
	*IL6R*	Interleukin 6 receptor	19003.48	4036.01	− 2.24	3.77e−07
	*KEAP1*	Kelch like ECH associated protein 1	1894.69	283.52	− 2.74	6.23e−07
	*MGST1*	Microsomal glutathione S− transferase 1	385.27	16.68	− 4.52	8.22e−03
	*PDGFA*	Platelet derived growth factor subunit A	29.57	150.10	2.37	1.98e−02
	*PDGFRB*	Platelet derived growth factor receptor beta	314.64	0	− 6.68	7.00e−03
	*PIK3R3*	Phosphoinositide− 3− kinase regulatory subunit 3	152.15	0	− 5.62	2.17e−02
	*RALB*	RAS like proto− oncogene B	7354.73	1417.61	− 2.38	4.91e−09
	*RALGDS*	Ral guanine nucleotide dissociation stimulator	959.68	16.68	− 5.85	4.33e−04
	*RASSF1*	Ras association domain family member 1	5456.91	717.14	− 2.93	1.40e−13
	*SOS1*	SOS Ras/Rac guanine nucleotide exchange factor 1	1679.24	6804.51	2.02	1.61e−10
	*TGFA*	Transforming growth factor alpha	951.89	50.03	− 4.25	2.30e−04
	*TXNRD3*	Thioredoxin reductase 3	18.50	133.42	2.78	1.63e−03
Proliferation	*APC*	APC regulator of WNT signaling pathway	1038.06	12174.73	3.55	1.22e−15
	*BRAF*	B− Raf proto− oncogene, serine/threonine kinase	1366.54	9206.1	2.75	1.26e−17
	*CCND2*	Cyclin D2	4458.14	66.71	− 6.06	2.79e−14
	*CCND3*	Cyclin D3	16147.13	3485.64	− 2.21	1.37e−06
	*CCNE2*	Cyclin E2	27.48	316.88	3.53	2.98e−03
	*FGFR2*	Fibroblast growth factor receptor 2	85.98	950.63	3.47	4.66e−03
	*FLT3LG*	fms related tyrosine kinase 3 ligand	92.37	0	− 4.90	4.48e−02
	*FZD1*	Frizzled class receptor 1	271.89	50.03	− 2.44	2.45e−02
	*FZD6*	Frizzled class receptor 6	64.00	517.01	3.01	1.78e−05
	*HMOX1*	Heme oxygenase 1	3398.05	733.82	− 2.21	1.22e−03
	*HRAS*	HRas proto− oncogene, GTPase	460.16	83.39	− 2.46	2.96e−03
	*IGF1R*	Insulin like growth factor 1 receptor	2771.65	11591.01	2.06	1.07e−03
	*KEAP1*	Kelch like ECH associated protein 1	1894.69	283.52	− 2.74	6.23e−07
	*LRP6*	LDL receptor related protein 6	55.66	1400.93	4.64	7.96e−06
	*MAPK3*	Mitogen− activated protein kinase 3	5762.75	1334.22	− 2.11	1.12e−04
	*MGST1*	Microsomal glutathione S− transferase 1	385.27	16.68	− 4.52	8.22e−03
	*PDGFA*	Platelet derived growth factor subunit A	29.57	150.10	2.37	1.98e−02
	*PDGFB*	Platelet derived growth factor subunit B	171.69	0	− 5.81	1.63e−02
	*PDGFRB*	Platelet derived growth factor receptor beta	314.64	0	− 6.68	7.00e−03
	*RB1*	RB transcriptional corepressor 1	1225.51	10390.22	3.08	8.52e−20
	*SOS1*	SOS Ras/Rac guanine nucleotide exchange factor 1	1679.24	6804.51	2.02	1.61e−10
	*TGFA*	Transforming growth factor alpha	951.89	50.03	− 4.25	2.30e−04
	*TXNRD3*	Thioredoxin reductase 3	18.50	133.42	2.78	1.63e−03
	*WNT10B*	Wnt family member 10B	229.22	0	− 6.22	9.03e−03
Sustained angiogenesis	*BRAF*	B− Raf proto− oncogene, serine/threonine kinase	1366.54	9206.10	2.75	1.26e−17
	*FGFR2*	Fibroblast growth factor receptor 2	85.98	950.63	3.47	4.66e−03
	*FLT3LG*	fms related tyrosine kinase 3 ligand	92.37	0	− 4.90	4.48e−02
	*HRAS*	HRas proto− oncogene, GTPase	460.16	83.39	− 2.46	2.96e−03
	*IGF1R*	Insulin like growth factor 1 receptor	2771.65	11591.01	2.06	1.07e−03
	*JAG1*	Jagged canonical Notch ligand 1	99.62	517.01	2.37	2.68e−04
	*MAPK3*	Mitogen− activated protein kinase 3	5762.75	1334.22	− 2.11	1.12e−04
	*NOTCH2*	Notch receptor 2	8983.16	39609.57	2.14	6.81e−07
	*PDGFA*	Platelet derived growth factor subunit A	29.57	150.10	2.37	1.98e−02
	*PDGFB*	Platelet derived growth factor subunit B	171.69	0	− 5.81	1.63e−02
	*PDGFRB*	Platelet derived growth factor receptor beta	314.64	0	− 6.68	7.00e−03
	*SOS1*	SOS Ras/Rac guanine nucleotide exchange factor 1	1679.24	6804.51	2.02	1.61e−10
	*TGFA*	Transforming growth factor alpha	951.89	50.03	− 4.25	2.30e−04
	*TGFB1*	Transforming growth factor beta 1	28839.34	6287.5	− 2.20	1.64e−04
Genomic instability	*BAD*	BCL2 associated agonist of cell death	826.17	116.74	− 2.82	5.74e−05
	*BBC3*	BCL2 binding component 3	1275.40	66.71	− 4.26	5.98e−06
Insensitivity to anti−growth signals	*SMAD2*	SMAD family member 2	2524.65	10957.26	2.12	3.49e−32
	*TGFB1*	Transforming growth factor beta 1	28839.34	6287.5	− 2.20	1.64e−04

We highlighted the transcripts mean counts obtained after DESeq2 normalization, the fold changes computed as log_2_ (MRS mean counts/Control mean counts) and the q-value for each of the DEGs in our analysis that take place in the most implicated process in KEGG map “Pathways in cancer”. All values are rounded to the second decimal digit

**Figure 3.**
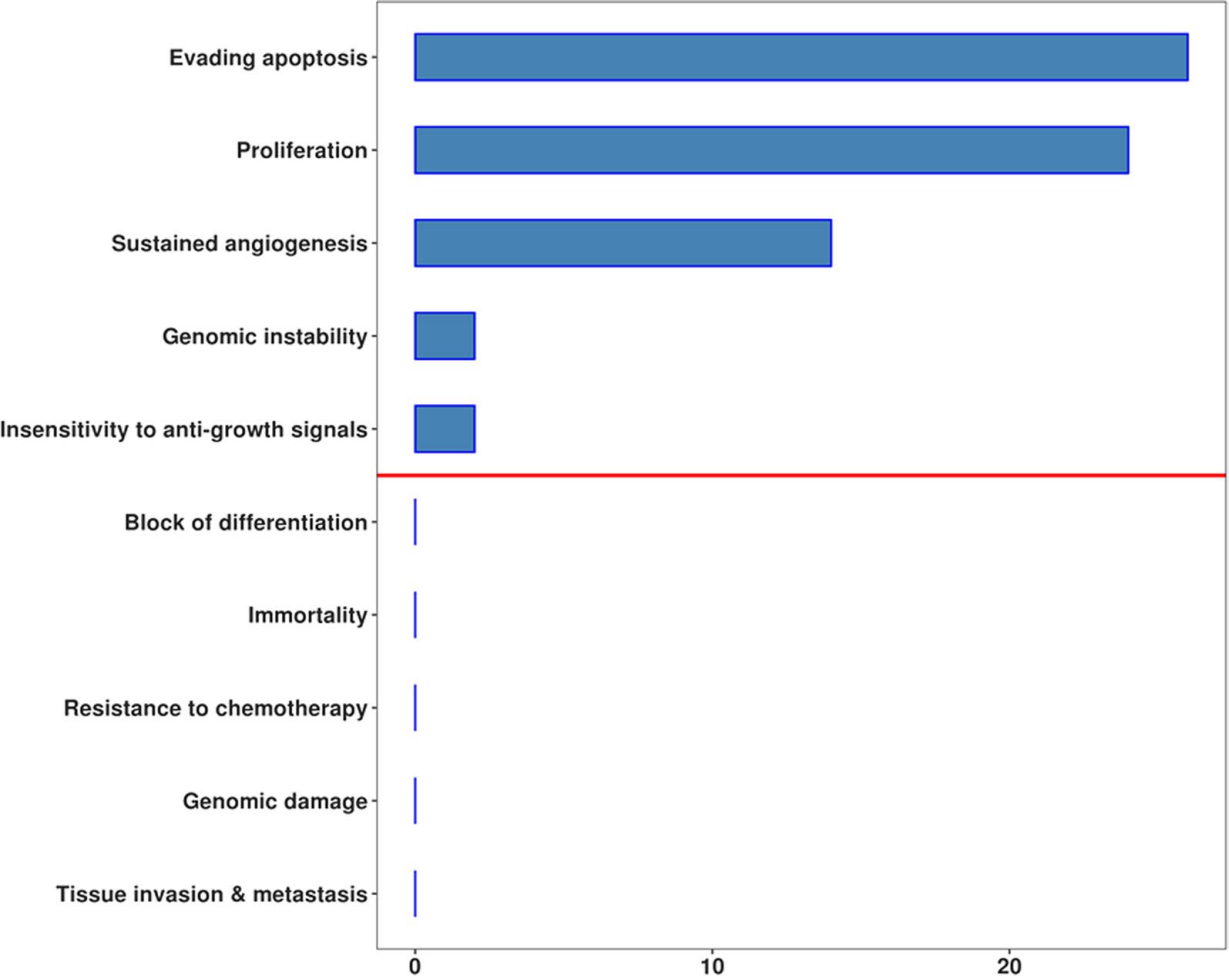
Number of DEGs in the signaling identified inside the KEGG map “Pathways in cancer”. The red line represents the median value so that “ERK signaling”, “PI3K signaling”, “JAK-STAT signaling”, “Calcium signaling”, “Other RAS signaling” and “WNT signaling” have a major implication in our study

In addition to the biological implications, we also inspected the signals and their related genes in cancer (Figure 3). The signals in which the most of the genes were implicated (Table 3) are “PI3K signaling” (*BAD, EGF, FGFR2, FLT3LG, HRAS, IGF1R, PDGFA, PDGFRB, PIK3R3, SOS1, TGFA*), “ERK signaling” (*BRAF, FGFR2, FLT3LG, HRAS, IGF1R, MAPK3, PDGFA, PDGFB, PDGFRB, SOS1, TGFA*), “JAK-STAT signaling” (*EGF, FLT3LG, IFNAR2, IL12RB1, IL13RA1, IL15, IL2RB, IL2RG, IL6R*), “calcium signaling” (*CAMK2D, EGF, PDGFA, PDGFB, PDGFRB, PRKCG, TGFA*), “other RAS signaling” (*EGF, HRAS, RALB, RALGDS, RASSF1, SOS1*), and “WNT signaling” (*APC, FZD1, FZD6, LRP6, WNT10B*).

**Table 3.** DEGs inside the KEGG map “Pathways in cancer” identified in the most implicated signalings

Signaling	Gene symbol	Gene name	Control mean counts	MRS mean counts	Fold change	q- value
PI3K signaling	*BAD*	BCL2 associated agonist of cell death	826.17	116.74	− 2.82	5.74 e−05
	*EGF*	Epidermal growth factor	62.50	700.46	3.49	2.01 e−04
	*FGFR2*	Fibroblast growth factor receptor 2	85.98	950.63	3.47	4.66 e−03
	*FLT3LG*	fms related tyrosine kinase 3 ligand	92.37	0	− 4.90	4.48 e−02
	*HRAS*	HRas proto− oncogene, GTPase	460.16	83.39	− 2.46	2.96 e−03
	*IGF1R*	Insulin like growth factor 1 receptor	2771.65	11591.01	2.06	1.07 e−03
	*PDGFA*	Platelet derived growth factor subunit A	29.57	150.10	2.37	1.98 e−02
	*PDGFRB*	Platelet derived growth factor receptor beta	314.64	0	− 6.68	7.00 e−03
	*PIK3R3*	Phosphoinositide− 3− kinase regulatory subunit 3	152.15	0	− 5.62	2.17 e−02
	*SOS1*	SOS Ras/Rac guanine nucleotide exchange factor 1	1679.24	6804.51	2.02	1.61 e−10
	*TGFA*	Transforming growth factor alpha	951.89	50.03	− 4.25	2.30 e−04
ERK signaling	*BRAF*	B− Raf proto− oncogene, serine/threonine kinase	1366.54	9206.10	2.75	1.26 e−17
	*FGFR2*	Fibroblast growth factor receptor 2	85.98	950.63	3.47	4.66 e−03
	*FLT3LG*	fms related tyrosine kinase 3 ligand	92.37	0	− 4.90	4.48 e−02
	*HRAS*	HRas proto− oncogene, GTPase	460.16	83.39	− 2.46	2.96 e−03
	*IGF1R*	Insulin like growth factor 1 receptor	2771.65	11591.01	2.06	1.07 e−03
	*MAPK3*	Mitogen− activated protein kinase 3	5762.75	1334.22	− 2.11	1.12 e−04
	*PDGFA*	Platelet derived growth factor subunit A	29.57	150.10	2.37	1.98 e−02
	*PDGFB*	Platelet derived growth factor subunit B	171.69	0	− 5.81	1.63 e−02
	*PDGFRB*	Platelet derived growth factor receptor beta	314.64	0	− 6.68	7.00 e−03
	*SOS1*	SOS Ras/Rac guanine nucleotide exchange factor 1	1679.24	6804.51	2.02	1.61 e−10
	*TGFA*	Transforming growth factor alpha	951.89	50.03	− 4.25	2.30 e−04
JAK− STAT signaling	*EGF*	Epidermal growth factor	62.50	700.46	3.49	2.01 e−04
	*FLT3LG*	fms related tyrosine kinase 3 ligand	92.37	0	− 4.90	4.48 e−02
	*IFNAR2*	Interferon alpha and beta receptor subunit 2	2464.19	83.39	− 4.89	6.69 e−11
	*IL12RB1*	Interleukin 12 receptor subunit beta 1	2509.33	617.08	− 2.02	1.76 e−05
	*IL13RA1*	Interleukin 13 receptor subunit alpha 1	5850.09	1184.12	− 2.30	2.81 e−05
	*IL15*	Interleukin 15	240.69	50.03	− 2.26	4.34 e−02
	*IL2RB*	Interleukin 2 receptor subunit beta	8781.96	733.82	− 3.58	8.80 e−08
	*IL2RG*	Interleukin 2 receptor subunit gamma	6894.04	33.36	− 7.69	1.12 e−11
	*IL6R*	interleukin 6 receptor	19003.48	4036.01	− 2.24	3.77 e−07
Calcium signaling	*CAMK2D*	Calcium/calmodulin dependent protein kinase II delta	1091.52	5470.29	2.32	6.63 e−11
	*EGF*	Epidermal growth factor	62.50	700.46	3.49	2.01 e−04
	*PDGFA*	Platelet derived growth factor subunit A	29.57	150.10	2.37	1.98 e−02
	*PDGFB*	Platelet derived growth factor subunit B	171.69	0	− 5.81	1.63 e−02
	*PDGFRB*	Platelet derived growth factor receptor beta	314.64	0	− 6.68	7.00 e−03
	*PRKCG*	Protein kinase C gamma	3.07	166.78	5.79	6.10 e−08
	*TGFA*	Transforming growth factor alpha	951.89	50.03	− 4.25	2.30 e−04
Other RAS signaling	*EGF*	Epidermal growth factor	62.50	700.46	3.49	2.01 e−04
	*HRAS*	HRas proto− oncogene, GTPase	460.16	83.39	− 2.46	2.96 e−03
	*RALB*	RAS like proto− oncogene B	7354.73	1417.61	− 2.38	4.91 e−09
	*RALGDS*	Ral guanine nucleotide dissociation stimulator	959.68	16.68	− 5.85	4.33 e−04
	*RASSF1*	Ras association domain family member 1	5456.91	717.14	− 2.93	1.40 e−13
	*SOS1*	SOS Ras/Rac guanine nucleotide exchange factor 1	1679.24	6804.51	2.02	1.61 e−10
WNT signaling	*APC*	APC regulator of WNT signaling pathway	1038.06	12174.73	3.55	1.22 e−15
	*FZD1*	Frizzled class receptor 1	271.89	50.03	− 2.44	2.45 e−02
	*FZD6*	Frizzled class receptor 6	64.00	517.01	3.01	1.78 e−05
	*LRP6*	LDL receptor related protein 6	55.66	1400.93	4.64	7.96 e−06
	*WNT10B*	Wnt family member 10B	229.22	0	− 6.22	9.03 e−03

We highlighted the transcripts mean counts obtained after DESeq2 normalization, the fold changes computed as log_2_ (MRS mean counts/Control mean counts) and the q-value for each of the DEGs in our analysis that take place in the most implicated signalings in KEGG map “Pathways in cancer”. All values are rounded to the second decimal digit

## Discussion

Homozygous mutations in the transcription factor (TF) *RFX6* are the cause of the MRS associating neonatal diabetes congenital digestive system defects, including biliary atresia, pancreatic hypoplasia, duodenal and/or jejunal atresia, intestinal malrotation, gallbladder aplasia, and cholestasis. In our case, heterotopic gastric mucosa in the small bowel tissue is also reported [2, 7, 8].

*RFX6* is included in the TF regulatory network of human pancreas development. During early pancreas specification and lineage commitment, specific TFs and other critical markers are expressed at each stage [9, 23]. Mutations in *RFX6* are involved in endocrine and exocrine pancreatic insufficiency and also in the altered maturation of the enteroendocrine cell subpopulation in the gastrointestinal tract, leading to diabetes and severe malabsorption [23]. Additionally, the *RFX6* gene controls genetic programs, regulating Peptidergic Enteroendocrine cell differentiation and identity. The constitutive inactivation of *RFX6* leads to a lack of functional compensation in some pluripotent endodermal stem cells, which consequently lose or do not acquire their proper regional identity, resulting in patchy patterns in gastric tissue [9].

Our network analysis highlighted *AGAP4*, *ATR, EHBP1, EIF2AK3, GHRL, GLIS3, GPR68, IER3IP1, IFT88, MAFB, PLAGL1, RFX7, RPGRIP1L,* and *SYTL4* as interactors of *RFX6* (Table 1). Among them, *EIF2AK3, IER3IP1, GLIS3, RFX7*, and *IFT88* were present to the highest degree (Fig. 1).

The interaction of *RFX6* with other upregulated genes, such as *EIF2AK3*, may induce a severe diabetic condition and could be related to multi-organ impairment and cancer degeneration. In fact, the *EIF2AK3* gene is also involved in the mechanism by which endoplasmic reticulum (ER) stress leads to programmed cell death (PCD) [24, 25]. PCD is an essential part of the maintenance of cellular homeostasis and survival of multicellular organisms during embryologic development, after birth, and during adulthood. Cell death is suspected to be also partially responsible for degenerative diseases and the uncontrolled proliferation of cancer cells [24, 25]. In heterotopic gastric mucosa, PCD and the vulnerability of surface mucosa to repetitive erosion and regeneration could be the cause of aberrations in the gastric mucosa and cancer degeneration at the surface mucosa [16, 26].

To date, in the literature less than twenty patients with MRS have been described and long-term follow ups of these patients are limited. Considering the reported risk of NCDs and cancer in patients with chromosomal CAs [18], the analysis of the transcriptomic profile may be useful to discover genetic alteration and support links to the disease. It is noteworthy that the analysis can be particularly useful when the individual has a known cancer predisposition, as in our patient who presented with heterotopic gastric mucosa.

*EIF2AK3* also interacts with *IER3IP1* and *GLIS3*. *IER3IP1* encodes the immediate early response 3 interacting protein 1 into the ER. It also participates in permanent neonatal diabetes mellitus and seems to alter the death and proliferation rate of the β-cells that potentially occur after ER stress [27]. Sun, J, and Ren, D., observed an alteration in *IER3IP1* expression with apoptotic proteins of the BCL-2 family [28], while the PI3K/AKT/mTOR pathway seems likely to play a key role in β-cell growth, proliferation, and survival [29]. Interestingly, in the PI3K signaling investigated in our study, we observed the downregulation of the BCL2-associated agonist of cell death encoded by the *BAD* gene along with the deregulation of *EGF, FGFR2, FLT3LG, HRAS, IGF1R, PDGFA, PDGFRB, PIK3R3, SOS1,* and *TGFA*. On the other hand, *GLIS3* belongs to the GLIS protein family of zinc finger and occurs in many diseases, including neonatal diabetes mellitus, aplasia, hypoplasia, hypothyroidism, growth retardation, atrial septal defects, autoimmune diseases, and neurological disorders. *GLIS3* deregulation was also associated with liver, thyroid, and breast cancer. In particular, breast carcinogenesis seems to take advantage of the WNT/B-catenin pathway [30]. The WNT signaling in our study is altered by the deregulation of *APC, FZD1, FZD6, LRP6*, and *WNT10B*, which are included by KEGG in the process of cancer proliferation.

*RFX6* also interacts with *IFT88* and *RFX7*, which in our network are present to a high degree and, in turn, interact each other.

Intraflagellar Transport 88, encoded by *IFT88*, is involved in cilium biogenesis and the genetic mutations carried on this gene were firstly associated with kidney disease. Then, cilia dysfunction was also linked to diabetes and cancer. Recently, *IFT88* was also suggested to play a role in the primary cilium in Hedgehog and WNT signaling [31]. Interestingly, its interactor *RPGRIP1L,* which encodes for RPGRIP1 Like, is localized in ciliated cells and seems to regulate the activity of the ciliary proteosome, which was observed to be altered in many cancers [32]. Contrary to other members of Regulatory Factor X family, *RFX7* is poorly characterized but shows a high tumor suppressor potential in lymphoid cancers at least [33, 34]. Furthermore, *RFX7* is upregulated in breast cancer but negatively correlates with metastatic development [35]. Additionally, *RFX6* and *RFX7* directly interact with *ATR*, encoding for ATR Serine/Threonine Kinase. ATR Serine/Threonine Kinase, along with its downstream Checkpoint Kinase 1, plays a crucial role in the regulation of the cell cycle for repairing DNA in response to damage. Similar to our analysis, other studies have shown that the activation of ATR leads to cell survival and proliferation; thus, several inhibitors of ATR/CHK1 have been proposed to treat cancer [28, 36].

Similar to *GLIS3*, the *PLAGL1* gene encodes for a zinc finger protein associated with diabetes and has been nominated as a tumor suppressor. It can handle the cell cycle as well as apoptosis and was found to be downregulated in many cancers, such as prostate, colon, ovarian, and breast [37]. The downregulation of *PLAGL1* in our analysis is in line with this observation, so its role in the cancer degeneration of heterotopic gastric areas cannot be excluded.

*MAFB* and *GPR68* genes are downregulated interactors of *RFX6* that Swiss-Prot associates with cancer. The MAF BZIP Transcription Factor B, encoded by *MAFB*, contributes to the differentiation of pancreatic α- and β-cells and to adult islet function. Nevertheless, it expresses proto-oncogene or tumor suppressor potential depending on the cell context. Lu et al. showed that MafB could play a pivot role in the proliferation of β-cells as well as in tumorigenesis condition [38]. The tumor suppressor gene *GPR68*, known as ovarian cancer G protein-coupled receptor 1 (OGR1), is a transmembrane receptor of the proton-sensing G protein-coupled receptors that is activated when extracellular levels of PH are altered. *RFX6* regulates the transcription of *GPR68* in adult human β-cells, in turn promoting the production of inflammatory interleukin 8 [39]. Herein, we observed the downregulation of *IL15* along with the receptors *IL2RB, IL2RG, IL6R, IL12RB1*, and *IL13RA1* in JAK-STAT signaling. In cancer, this culminates with the evading apoptosis event, as reported in KEGG. Additionally, the signal could be also implicated in the development of eosinophilic colitis. The *GHRL* gene, encoding ghrelin and obestatin prepropeptide, is associated with insulin secretion and downregulated. Our research group has already observed in a previous work that an infant with VACTERL and esophageal atresia carried a missense mutation on *GHRL* linked to metabolic syndrome [40]. In addition, the role of *GHRL* seems to be crucial in cell proliferation, migration, and invasion, as well as in inflammation in many cancers. Even so, its role as a promoter or inhibitor is still up for debate [41]. *SYTL4* encodes for the Synaptogamin Like 4, which is localized on the microtubule cytoskeleton. Liu et al. showed that Synaptogamin Like 4 hinders microtubule polymerization, reducing the stability. Furthermore, high levels of *SYTL4*, as in our case study, are associated with a poor prognosis for breast cancer [42]. *EHBP1* encodes EH Domain Binding Protein 1 and is poorly characterized. Nevertheless, *EHBP1* has already been associated with cancer and is required for the insulin-mediated translocation of glucose transporter type 4 [43, 44]. *AGAP4* encodes for ArfGAP with GTPase domain and Ankyrin Repeat and PH Domain 4, but very little is known about it. It is part of the family of centaurins as proteins with a GTPase-like domain which are known to regulate cell proliferation and vesicular trafficking. For similarity, GTPase-like centaurin *γ* − 1 is associated with cancer, in which it promotes cell invasion and prevents apoptosis [45].

In our analysis, we also wanted to further inspect the different hallmarks of cancer, as well as which of them could allow its development [46]. We categorized each process based on the amount of DEGs involved (Table 2). Thus, we found that “evading apoptosis”, “proliferation”, “sustained angiogenesis”, “genomic instability”, and “insensitivity to anti-growth signals” are the most relevant adopted strategies (Fig. 2). Noteworthy, research on cancer has revealed many pathways that are able to promote these processes (Fig. 3). For this reason, we inspected the key signals in MSR and we observed that “ERK signaling”, “PI3K signaling”, “JAK-STAT signaling”, “calcium signaling”, “other RAS signaling”, and “WNT signaling” show the highest involvement (Table 3). Notably, the PI3K, Wnt/β-catenin, and RAS/ERK signaling pathways can handle cellular metabolism and consequently influence signal transduction and oxidative stress potential [47]. Furthermore, cancer proliferation and invasiveness were reported to be caused by altered calcium signaling in the tumor microenvironment [48]. The regulation of the microenvironment can also be changed through the alteration of Jak-Stat signaling. This signaling is mediated by inflammatory cytokines that promote the self-renewal of cancer stem cells and differentiation [48, 49].

## Conclusions

MRS is caused by the mutation of the *RFX6* gene and is characterized by neonatal diabetes, pancreatic hypoplasia, intestinal atresia, and gallbladder hypoplasia or aplasia and chronic diarrhea. A constitutive inactivation of *RFX6* may also lead to gastric heterotopia. In our MRS patient, we signaled the interactors of *RFX6* with other up- and downregulated genes that may be related to severe diabetic condition, multi-organ impairment, and cancer predisposition. Notably, many dysregulated genes take place in the mechanisms of evading apoptosis, proliferation, sustained angiogenesis, genomic instability, and insensitivity to anti-growth signals, which may lead to triggering carcinogenesis. The possibility of the patient developing cancer degeneration in heterotopic gastric mucosa and/or additional long-term tumoral sequelae is not excluded. Personalized prevention and treatment strategies should be proposed.

## Methods

### Patient

The patient is a male infant born to consanguineous Pakistani parents at 37 weeks+2 days of gestation, with intrauterine growth restriction (weight 1417 g, <3rd percentile; length 41 cm, < 3rd percentile; head circumference 34 cm, 75th percentile). The mother was diagnosed with hyperthyroidism during pregnancy, with a normal glucose profile. The father is diabetic. A prenatal suspicion of duodenal atresia was posed.

He scored an APGAR of 1 and 8 at 1 and 5 minutes, respectively, requiring non-invasive ventilation during the first 24 hours. At birth, the patent foramen ovale and duodenal atresia were detected. The baby underwent surgical repair of the type III duodenal atresia. At operation, gallbladder malposition, hypoplasia, an ectopic pancreas, and duodenal heterotopic gastric mucosa were found. Histologic evaluation confirmed the duodenal presence of gastric mucosa without signs of erosion.

The infant started insulin infusion for hyperglycemia from day 1; during a hyperglycemic episode, his C-peptide level was very low (< 0.1 ng/mL, nv 0.8–4.2 ng/mL).

From day 8, watery diarrhea became evident and persistent, associated with failure to thrive and dependency on parenteral nutrition.

The infant underwent genetic analysis, which detected homozygous missense mutations not reported previously in the RFX6 [p.Ser500Gly (c.1498A>G)], and MRS was diagnosed. A heterozygous mutation was detected in the parents.

Neonatal diabetes was confirmed and insulin pump therapy and continuous glucose monitoring were started on day 40.

At the age of 2 months, bilateral hyperechogenic renal parenchyma with normal renal function was detected.

During monitoring, protracted diarrhea persisted despite several interventions, including dietary adaptations with semi-elemental, elemental, and low long-chain triglyceride formulas and the use of pancreatic enzymes. Recurrent hospitalization for sepsis was recorded. Metabolic control of diabetes was near-optimal.

Stool was intermittently positive for microscopic blood, and iron substitution was required for chronic anemia. At the age of 8 months, the anemia worsened and repeated blood transfusions were required. Endoscopic investigation confirmed duodenal ectopic gastric mucosa including the entire duodenal surface. An extensive jejunal gastric heterotopia was also recorded, and histologic evaluation confirmed the jejunal heterotopic gastric mucosa with signs of intestinal mucosa erosion. An eosinophilic colitis was also confirmed. Total parenteral nutrition was introduced. Progressively, the boy started to improve clinically and we began a reintroduction of elemental, high medium-chain triglyceride formula by the mouth, with the use of pancreatic enzymes and multiple vitamin supplements associated with parental nutrition. Diarrhea persisted and consisted of 5 to 8 watery stools per day. At 13 months, antihypertensive treatment was started.

Currently, the patient is 15 months years old, 71.5 cm in length (< 3rd percentile), and with a 7835 kg body weight (< 3rd percentile). He shows an unstable control of diabetes, with high glycemic variability and a HbA1c 7.3% (56.3 mmol/mol). In addition to personalized parental nutrition, a hypoallergenic diet (using an elemental formula in milk) with pancreatic enzymes and multiple vitamin supplementation was adopted.

### Transcriptomic analysis

The use of a control group is necessary in order to inspect the genes differentially expressed in MRS. From the Sequence Read Archive (SRA) [51], we selected the runs of the healthy samples GSM2370017, GSM2370185, GSM2370217, GSM2370225, GSM2370231, GSM2370237, GSM2370251, GSM2370261, GSM2370269, and GSM2370271 that belong to the bioproject PRJNA352062 [50].

MRS and control samples were analyzed, taking advantage of the same workflow. Fastq raw data were inspected with fastQC in order to analyze their quality. We toke advantage of Trimmomatic (version 0.38, Usadel Lab, Aachen, Germany) [51] to drop the poor-quality bases and potential adapters. Then, the Spliced Transcripts Alignment to a Reference (STAR) RNA-seq aligner [52] was used to align and sort the reads against the reference homo sapiens GRCh38 genome. The python package htseq-count counted the number of transcripts in each region [53]. The package DESeq2 of Bioconductor found the genes that were differentially expressed (DEGs) between MRS and the control group using the R programming language [54]. The Benjamini–Hochberg post hoc test was used to remove the false positives to correct the *p*-value. All the genes whose *q*-value was lower than 0.05 and whose fold change was lower than − 2.0 or over 2.0 were kept.

To highlight oncogene and tumor suppressor genes in our analysis, we observed the DEGs that take place in the KEGG [55] map “pathways in cancer” from homo sapiens (hsa05200). Indeed, this map collects the different signaling pathways that are activated in cancer and the different biological processes that they trigger. Specifically, we observed which DEGs were included in the map, which biological processes they promote, and in which pathways they play a role. Furthermore, in order to study the behavior of the proteins that are known to interact with the RFX6 gene, we used the STRING [56] database. Thus, we input our DEGs along with *RFX6* and kept only the genes that transcribed proteins known to be direct interactors. Finally, we took advantage of the manual curated Swiss-Prot [57] database to provide a role of each DEG identified by STRING as an *RFX6* interactor. In detail, the Swiss-Prot “Keyword – Disease” section was inspected and the involvement of the interactors with unhealthy status was further explored in the literature.

## Data Availability

Not applicable.

## Abbreviations

MRS: Mitchell–Riley syndrome
CAs: Congenital anomalies
NCDs: Noncommunicable diseases
PCD: Programmed cell death
SRA: Sequence read archive
DEGs: Differentially expressed genes
